# A Spectroscopic and In Silico Description of the Non-Covalent Interactions of Phthalic Acid Imide Derivatives with Deoxyribonucleic Acid—Insights into Their Binding Characteristics and Potential Applications

**DOI:** 10.3390/molecules29225422

**Published:** 2024-11-17

**Authors:** Aleksandra Marciniak, Edward Krzyżak, Dominika Szkatuła, Krystian Mazurkiewicz, Aleksandra Kotynia

**Affiliations:** 1 Department of Basic Chemical Sciences, Faculty of Pharmacy, Wroclaw Medical University, Borowska 211a, 50-556 Wrocław, Poland; edward.krzyzak@umw.edu.pl (E.K.); aleksandra.kotynia@umw.edu.pl (A.K.); 2 Department of Medicinal Chemistry, Faculty of Pharmacy, Wroclaw Medical University, Borowska 211a, 50-556 Wrocław, Poland; dominika.szkatula@umw.edu.pl; 3 “Biomolecule” Student Science Club, Department of Basic Chemical Sciences, Faculty of Pharmacy, Wroclaw Medical University, Borowska 211a, 50-556 Wrocław, Poland; krystian.mazurkiewicz@student.umw.edu.pl

**Keywords:** phthalimide analog, spectroscopy, UV-Vis, CD, molecular modeling, DNA, interaction

## Abstract

The treatment of cancer represents one of the most significant challenges currently facing modern medicine. The search for new drugs that are effective in the treatment of patients is an ongoing endeavor. It is frequently the case that the molecular target of anticancer drugs is a DNA molecule. The therapeutic effect of a drug is achieved by influencing the structure of a macromolecule or by inhibiting its function. Among the synthetic substances with potential anticancer effects, particular attention should be paid to phthalic acid imide derivatives. Three phthalimide derivatives are employed in the treatment of multiple myeloma: thalidomide, pomalidomide, and lenalidomide. Nevertheless, the search for new derivatives with a diverse range of biological activities is ongoing. In light of the above, the subject of our investigation is four non-toxic phthalic acid imide derivatives. The objective was to analyze the interaction of these compounds with DNA. The use of spectroscopic and in silico methods has enabled us to demonstrate that all of the tested analogs can act as ligands for deoxyribonucleic acid, forming non-covalent bonds with it. All four compounds tested interact with the ctDNA molecule, binding in its minor groove. The most stable complex is formed here between deoxyribonucleic acid and the C derivative, in which the -CF_3_ group is attached to the benzene ring. What is interesting and important, the described mechanism of action is analogous to that observed between ctDNA and thalidomide, pomalidomide, and lenalidomide.

## 1. Introduction

Phthalimide derivatives are a group of heterocyclic compounds characterized by the presence of a -CO-N(R)-CO- fragment and an imide ring. This makes them an attractive group of compounds, useful for their use in medicine [1]. Three phthalimide analogs have been approved by the US FDA: thalidomide, lenalidomide, and pomalidomide [2]. Because of their broad, proven spectrum of activity, this group of compounds has been of great interest to researchers for many years. On the basis of the PubMed database, there was a dramatic increase in interest in this group of molecules after the year 2000. The search continues for new derivatives that show activity useful in pharmaceutical science. In the present era, considerable emphasis is being placed on the search for novel anticancer pharmaceutical agents. In our previous research, we investigated a series of novel analogs of phthalic acid imide [3,4]. It was demonstrated that these non-toxic compounds possess anti-inflammatory and analgesic properties. The COX-2/COX-1 value for compounds **B** and **D** was found to be higher than that observed for meloxicam. Moreover, all phthalic acid imide derivatives demonstrated both ROS and NO scavenging activity. The in vitro results were also confirmed by in silico methods [4]. Additionally, some of the tested compounds have been proven to have analgesic and anti-inflammatory effects using in vivo tests [5]. Furthermore, it was shown that these compounds form stable complexes with plasma proteins, which could positively influence their biodistribution [3]. Given the promising properties observed, further analysis was deemed necessary.

The analysis of the interaction between new anticancer drugs and the DNA molecule has been a significant aspect of the search for new drugs for many years. This is attributable to the fact that the DNA molecule represents a common molecular target for these compounds [6]. Small molecules can interact with deoxyribonucleic acid in several ways, but among the non-covalent interactions, three mechanisms are dominant: intercalation, groove binding, and electrostatic interactions [7]. All three phthalimide derivatives used in medicine: thalidomide, pomalidomide, and lenalidomide were also tested in this aspect [8,9,10]. It has been demonstrated that all of these drugs interact with the DNA molecule by binding to its minor groove.

**Figure 1:**
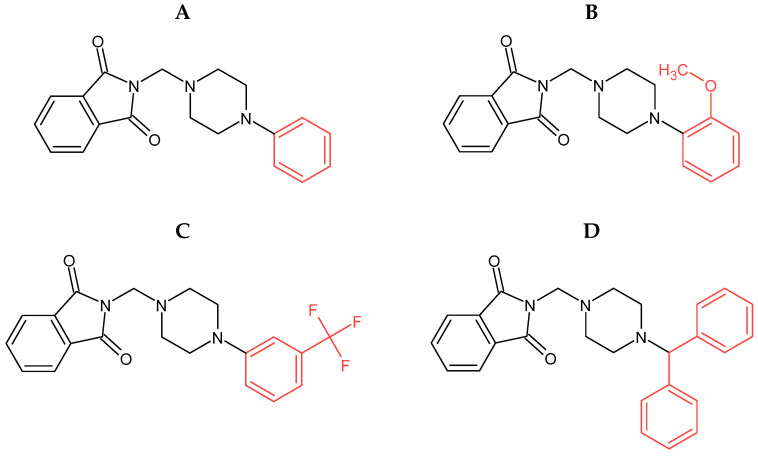
The structures of analyzed phthalic acid imide derivatives (**A**–**D**).

We, therefore, decided to also analyze our four promising phthalic acid imide derivatives **A**, **B**, **C**, and **D** (Figure 1) for their interaction with deoxyribonucleic acid. The structure of the compounds we studied was designed based on the structure of thalidomide, which has anticancer activity. It can, therefore, be assumed that the derivatives we analyzed could be characterized by similar properties, hence, the choice of ctDNA as a molecular target. Spectroscopic methods are a valuable tool in this field of research [7]. These methods combined with quantity analysis allow us to determine binding parameters, target selectivity, binding affinity, structure–activity relationships, and mechanism of action. This study presents the characterization of the non-covalent interactions of four phthalic acid imide derivatives with the ctDNA (calf thymus DNA).

We used a number of spectroscopic methods, such as UV-Vis, fluorescence, and circular dichroism spectroscopy. The monitoring of changes in absorption spectroscopy in the UV wavelength range let us calculate the apparent association constants (K) of equilibrium reaction, stoichiometry of binding, and changes in standard Gibbs free energy (ΔG). Fluorescence spectroscopy is also a significant analytical tool. It specifies the type of binding mechanism, whereas circular dichroism spectroscopy enables the verification of the impact of the binding process on disturbances in the spatial structure of DNA. Moreover, the aforementioned information can be corroborated and validated through the utilization of molecular docking. This in silico tool additionally hints at the specific atoms that are engaged in the interaction, and distance between them and allows us to determine the binding affinity energy.

## 2. Results and Discussion

### 2.1. Spectroscopic Studies

#### 2.1.1. UV-Vis Spectroscopy

**Figure 2:**
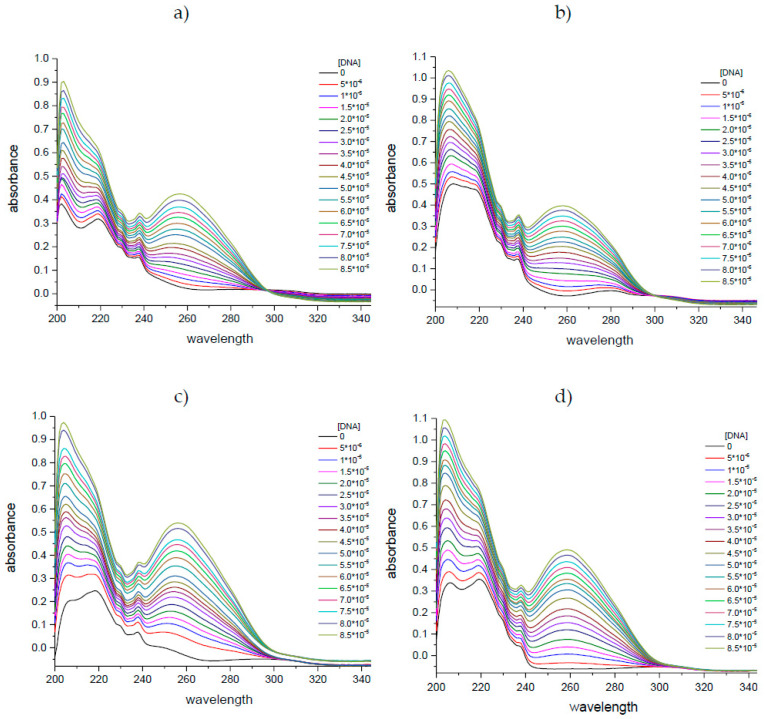
The absorption spectra in the absence and presence of various molar concentrations (M) of ctDNA added to phthalic acid imide derivative solutions (10 μM): (**a**) compound **A**, (**b**) compound **B**, (**c**) compound **C**, and (**d**) compound **D**.

**Figure 3:**
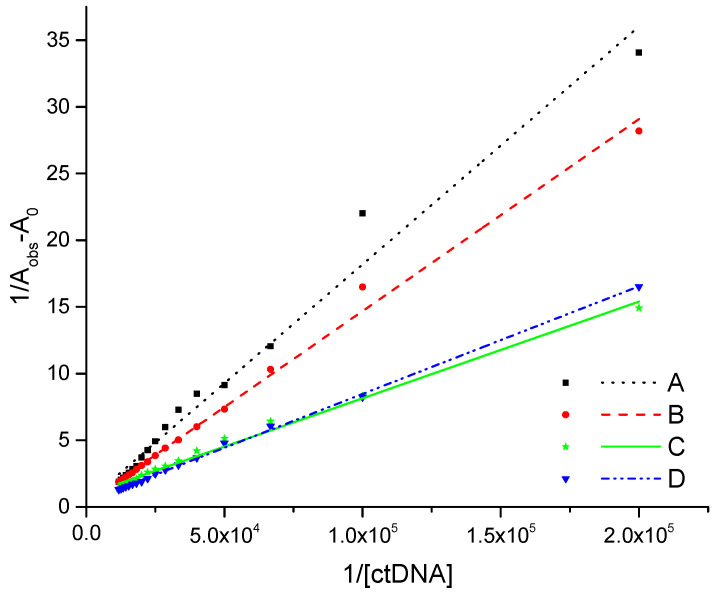
The UV data fitting line to the Benesi–Hildebrand equation. The absorption changes assumed by the addition of ctDNA to phthalic acid imide derivative solutions (10 μM): compound **A** (black, dotted line), compound **B** (red, dashed line), compound **C** (blue, dash-dotted line), and compound **D** (green, solid line).

Initially, UV absorption spectra were conducted with varying concentrations of ctDNA to facilitate a deeper understanding of the binding process. The UV spectra changes after ctDNA addition-induced hyperchromisity in all the tested compounds (Figure 2). The hyperchromic effect has been linked to the existence of synergistic non-covalent interactions, including electrostatic external contact, groove surface binding in conjunction with the helix, and hydrogen bonding [11,12]. The percentage of this effect for **A** and **B** derivatives is more than 50% whereas for **C** and **D** compounds is more than 70% (Table 1). This process culminates in the formation of complexes between phthalic acid imide derivatives and deoxyribonucleic acid (ctDNA). In addition, a small bathochromic shift in the maximum absorption band has been observed, but it is only 1 to 3 nm (Figure 2). The slight shift in the absorption spectra is connected with groove binding or electrostatic interaction while large changes in the position of the absorption maximum result from intercalation [13,14,15]. A quantitative measure of the equilibrium constant K for the binding of the tested compound to ctDNA was calculated using the Benesi–Hildebrand method [16]. The apparent association constants (K) for complex formation may be estimated from changes in absorbance at a fixed wavelength using a double reciprocal Equation (1) (described in Section “Materials and Methods”). Figure 3 presents the fitting results. The parameters of linear regression analysis along with the coefficient of determination (R^2^) are presented in Table 1. The dimension of the apparent association constants gained is adequate for the non-intercalative binding of ligands with ctDNA. The classical intercalators stand out in quite high binding constants in the range from 10^5^ to 10^11^ dm^3^·mol^−1^ [17]. In this study, the apparent association constants (K) range from 1.80 × 10^3^ to 1.28 × 10^4^ dm^3^·mol^−1^, suggesting a groove-binding mechanism (Table 2). Comparing the binding constant values of the tested derivatives, it can be concluded that compounds **C** and **D** interact more strongly with the ctDNA than **A** and **B**. This may be related to the chemical nature and size of the substituent in the N-arylpiperazine ring. Therefore, it can be assumed that substituents 1-(3-trifluoromethphenyl)piperazine (**C**) and 4-benzhydrylpiperazine/4-diphenylmethyl-1-piperazine (**D**) exhibited stronger interaction effects in the DNA groove pocket, followed by derivatives 1-(2-methoxyphenyl)piperazine (**B**) and 1-phenylpiperazine (**A**). The binding constants for thalidomide and its amino equivalent pomalidomide, used to treat multiple myeloma and other tumor, are of the same order of magnitude: 3.06 × 10^3^ and 8.73 × 10^3^ M^−1^, respectively. These drugs have the same 2,6-dioxo-piperidin-3-yl group as the compounds under investigation and have been assigned to a groove-type binding mechanism [8,9].

The characterization of the drug–DNA association reactions by investigating the thermodynamics of these processes is essential for a complete understanding of the mechanism of action. In addition, the van’t Hoff Equation (2) (described in Section “Materials and Methods”) was used to calculate the standard Gibbs free energy (ΔG). The negative values of ΔG prove that the complex formation between all four **A–D** phthalic acid imide compounds is a spontaneous process in experimental conditions (Table 2). Ihtshamul Haq noted that for groove binders, binding is largely due to the hydrophobic effect [18]. Juan S. Jiménez and co-authors analyzed ∆G values for a set of 3000 macromolecule/ligand affinities. For about 70% of the cases analyzed, ∆G is in the range of −46 to −26 kJ/mol [19]. The values obtained in this work are also in this range. In addition, previously known anticancer drugs such as thalidomide, pomalidomide, and lenalidomide, which interact with DNA in a groove manner, have a ∆G value of the binding reaction equal to −19.87, −22, and −29.45 kJ·mol^−1^, respectively. This is in good agreement with the values obtained for the phthalimide derivatives studied [8,9,10].

Summarizing the UV-Vis results described above, it can be concluded that the tested phthalimide derivatives interact with the DNA molecule via the groove-binding mechanism.

#### 2.1.2. Circular Dichroism Spectroscopy

**Figure 4:**
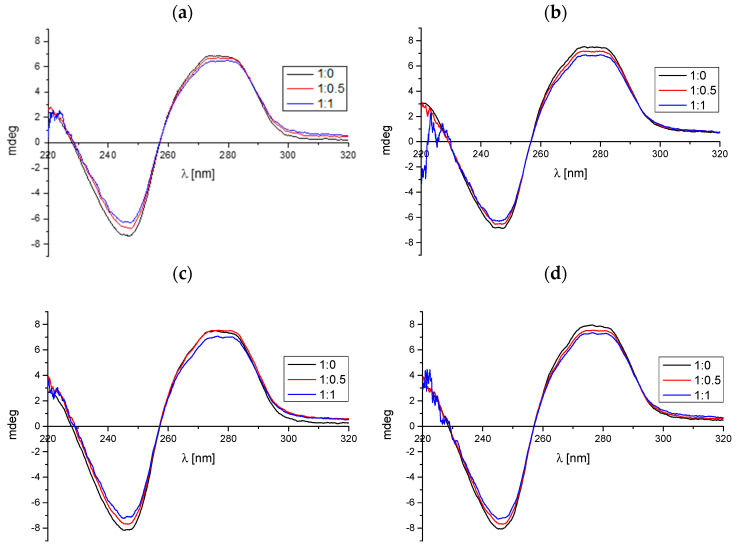
The CD spectra of ctDNA with increasing the concentration of the tested compounds: (**a**) compound **A**, (**b**) compound **B**, (**c**) compound **C**, and (**d**) compound **D**. The measurements were performed for the following molar ratios of ctDNA to phthalimide analog: 1:0, 1:0.5, and 1:1.

Another analytical method employed was circular dichroism spectroscopy, which enables the monitoring of the spatial structure of macromolecules during reactions with smaller ligands. In the case of ctDNA molecule spectra, two characteristic bands are observed as follows: negative between 240 and 250 nm, and positive near 280 nm. The first one is responsible for stacking interaction between the base pairs while the second one is the result of helicity strands [20]. This particular spectrum pattern, which was also identified in our measurements (Figure 4), is a distinctive feature of the B form of deoxyribonucleic acid (DNA) [21].

In the CD measurement, the tested compounds were introduced in incremental quantities to the ctDNA-containing sample to achieve equimolar concentrations of the reagents.

The previously published literature indicates that the mechanism of interaction in the studied system can be determined based on the changes observed in the course of CD spectra during reactions between the ctDNA molecule and the ligand. In the context of intercalation, notable differences are evident in the spectral appearance—both the position of the bands and their intensity undergo alteration. Nevertheless, in the instances of binding within the minor groove or as a consequence of electrostatic interactions, the alterations observed in the spectra are relatively small [22,23,24]. In the case of the tested compounds **A–D**, only minor alterations in the intensity of the bands characteristic of the DNA molecule are observed with an increase in their concentration (Figure 4, Table 3). The addition of each of the phthalic acid imide derivatives resulted in a slight alteration to the course of the spectra. It can, thus, be concluded that the analyzed compounds bind with deoxyribonucleic acid via the groove-binding mechanism, or alternatively, via electrostatic interactions. The outcome is in accordance with the findings derived from the UV-Vis data.

#### 2.1.3. Fluorescence Spectroscopy

*Investigation of the occurrence of electrostatic interactions in the tested systems*

**Figure 5:**
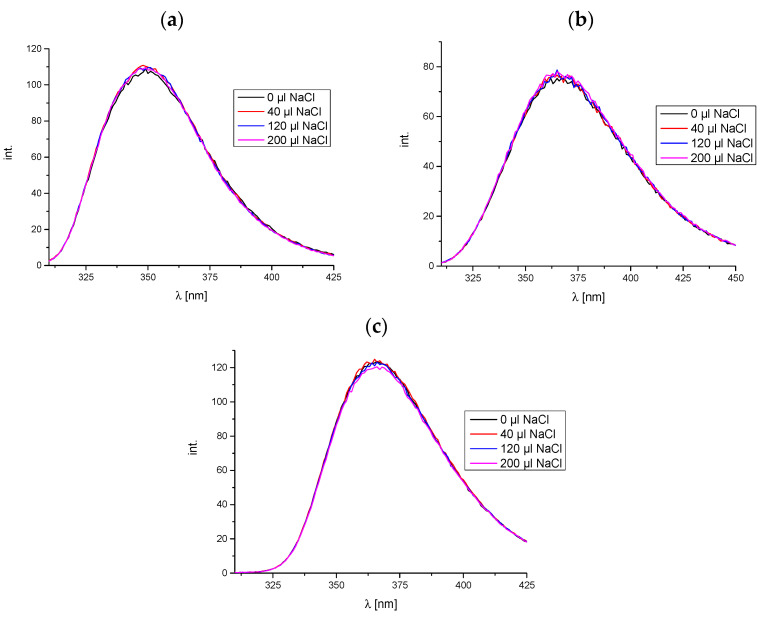
The emission spectra of (**a**) compound **A**, (**b**) compound **B**, and (**c**) compound **C** with ctDNA (1:1 reagents molar ratio) with the increasing NaCl concentration.

In order to investigate whether electrostatic interactions occur between the DNA molecule and phthalic acid imide derivatives, the fluorescence spectroscopy method was used. In this case, the changes occurring in the emission spectra of the tested compounds are analyzed during increasing ionic strength in the phthalimide derivative/ctDNA system. The increase in ionic strength was obtained by adding portions of 1 mM NaCl solution to the tested sample. The measurements were performed for compounds **A**, **B**, and **C**. It was not possible to perform this experiment for the D/ctDNA system, because this derivative does not show the fluorescence phenomenon, which was proven and described in the work [3]. The obtained spectra are shown in Figure 5.

Figure 5 illustrates that the intensity of the emission spectra remains unaltered when the ionic strength is increased in the three analyzed systems. This suggests the absence of any electrostatic interactions [25].

*Competitive binding studies with markers*

To confirm a specific mechanism or binding site in macromolecules, a convenient and effective method of analysis is to use markers with a known binding mode. In this study, we employed a similar experimental approach. Acridine orange was selected for the study. This molecule is a typical intercalator for ctDNA [7].

**Figure 6:**
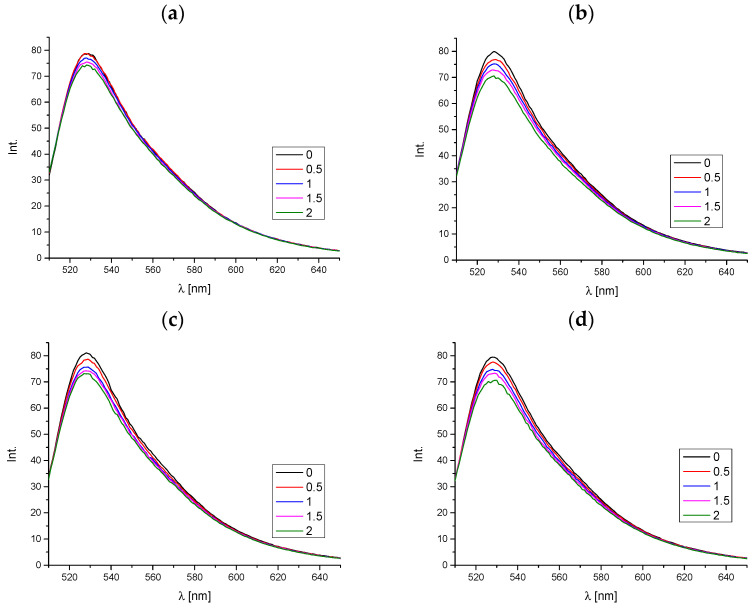
The emission spectra of ctDNA–acridine orange complex with an increase in the concentration of the tested compounds: (**a**) compound **A**, (**b**) compound **B**, (**c**) compound **C**, and (**d**) compound **D**. The measurements were performed for the following molar ratios of ctDNA to phthalimide analog: 1:0, 1:0.5, 1:1, 1:1.5, and 1:2.

Figure 6 shows the emission spectra of the complex between acridine orange and ctDNA. The marker demonstrates elevated emission levels in a complex with DNA relative to those observed in the absence of DNA [26,27]. This property allows for the assessment of whether the tested compounds are capable of displacing AO from its connections with deoxyribonucleic acid, thereby confirming the intercalation mechanism of binding. Based on the obtained results, it is also possible to calculate the percentage of AO replacement by the tested phthalimide derivatives using Equation (3) (described in Section “Materials and Methods”).

Upon recording the spectra at the excitation line of 480 nm and during the increase in the concentration of the tested phthalimide derivatives, a slight decrease in fluorescence intensity was observed (Figure 6). The highest percentage of AO exchange by the tested compounds was observed for derivative **B**; however, this value is still low and does not exceed 12% (Table 4). Consequently, it can be concluded that the tested mechanism of interaction through intercalation is not dominant in this case. Therefore, binding in the minor groove of DNA may be suspected here.

### 2.2. Molecular Modeling Study

**Figure 7:**
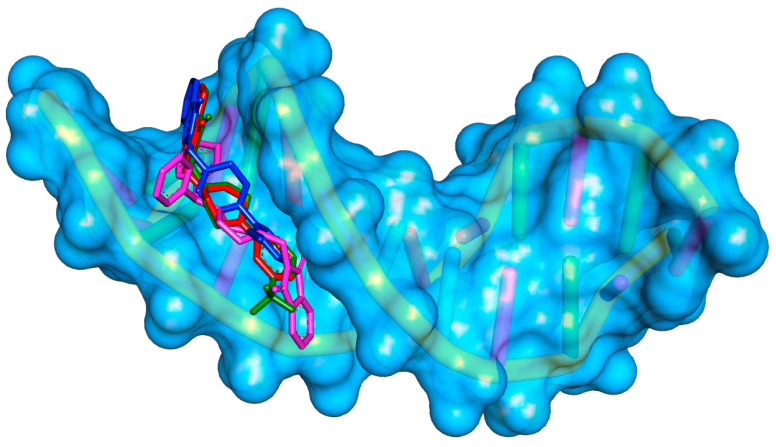
The pose of compounds **A** (red), **B** (blue), **C** (green), and **D** (pink) in the DNA minor groove.

**Figure 8:**
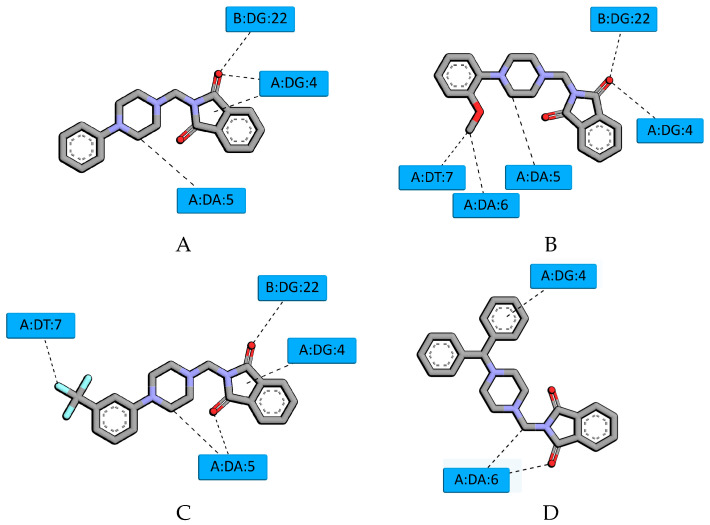
The 2D plot of the interactions between (**A**–**D**) and DNA.

**Figure 9:**
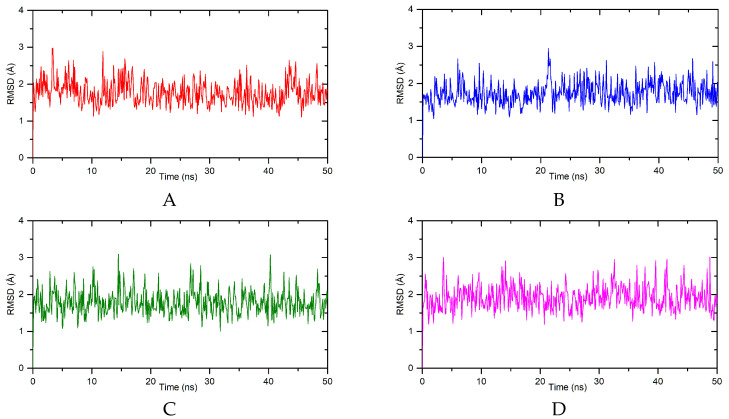
The RMSD plots of the DNA backbone atoms for systems with compounds **A–D**.

To determine the nature of the interaction of the tested compounds with DNA and to confirm the results from spectroscopic methods, in silico studies were conducted. The B-DNA crystal structure with ID 1vzk was selected for docking. This choice was made mainly because this crystal structure contains a ligand with a structure similar to the studied compounds, which interacts with DNA in the groove. Furthermore, in several works on interactions with DNA, this structure was chosen to explain the mechanism of interaction [28,29,30,31,32,33,34,35]. First, molecular docking was performed. For all the systems, the preferred site of interaction is a minor groove. The binding affinity was found as −8.5 kcal/mol, −8.4 kcal/mol, −8.7 kcal/mol, and −7.4 kcal/mol for **A**, **B**, **C**, and **D**, respectively. This suggests the formation of a stable complex with DNA. Figure 7 shows the pose of derivatives **A–D** in the minor groove of DNA, and Figure 8 shows the mode of interaction. All the systems are stabilized by hydrogen bonds. Conventional hydrogen bonds are formed between the oxygen of the carbonyl group of the phthalimide moiety and guanine DG22 (for **A**, **B**, **C**) and guanine DG4 (for **A** and **B**). The oxygen atom of the carbonyl group can also interact with adenine via a carbon–hydrogen bond (for **C** and **D**). The piperazine ring is involved in a carbon–hydrogen bond with adenine DA5 (for **A**, **B**, **C**). The substituent in the benzyl ring interacts with thymine DT7 and adenine DA6 forming carbon–hydrogen bonds (for **C**) or with thymine DT7 (for **C**). For the DNA-D complex, an interaction π-donor hydrogen bond type is formed. From the interactions we found two moieties of molecule favoring stabilization in the minor groove: the phthalimide group and the piperazine one. The presence of carbonyl allows the formation of hydrogen bonds. The piperazine moiety enables the formation of carbon–hydrogen bonds. The substituent in the benzyl ring also helps to stabilize the system. The binding affinity values (as well as the binding free energy) may also suggest that the presence of a large spherical group in the structure of compound **D** (dibenzyl) is not advantageous. Next, to confirm the stability of the complexes and determine the binding free energy, a molecular dynamics simulation method was used. In Figure 9 RMSD (root mean square deviation), the plot of the DNA backbone during 50 ns simulations is shown. All the systems behave in a similar manner. The average values were found as 1.77 ± 0.33 Å, 1.74 ± 0.31 Å, 1.79 ± 0.33 Å, and 1.92 ± 0.33 Å for **A–D**, respectively. The amplitude of the fluctuation is about 1 Å. Such results indicate the stable conformation of the complex. The predicted binding free energy was calculated to be −12.99 ± 0.65 kcal/mol, 14.64 ± 0.93 kcal/mol, 12.64 ± 0.90 kcal/mol, and 9.09 ± 1.04 kcal/mol for the A-D/DNA systems, respectively.

In summary, the applied in silico methods confirm the obtained experimental data. The tested phthalic acid imide derivatives interact with the DNA molecule in the same way as the drugs from this group described in the literature, binding in the minor groove.

## 3. Materials and Methods

### 3.1. Chemicals and Sample Preparation

The measurements were conducted in a water solution with phosphate-buffered (PBS) saline (Sigma-Aldrich Chemie GmbH, St. Louis, MO, USA); the concentration was 0.01 M and pH 7.4. The calf thymus deoxyribonucleic acid sodium salt (ctDNA) (Sigma-Aldrich Chemie GmbH, St. Louis, MO, USA) was dissolved in PBS. The stock solution concentration and purity were determined by the spectrophotometric method [36]. The ctDNA was free from protein and the final concentration was equal to 3.5 mM. The studied phthalimide derivatives (**A**–**D**) were dissolved in ethanol (Sigma-Aldrich Chemie GmbH, St. Louis, MO, USA), and the stock solution concentration was 1 mM. The tested phthalimide compounds were synthesized at the Wroclaw Medical University and were described in our previous work [4]. The solution was kept in the dark at 4 °C.

Chloride sodium (NaCl) (Avantor Performance Materials Poland S.A., Gliwice, Poland) and acridine orange hydrochloride hydrate (Sigma-Aldrich Chemie GmbH, St. Louis, MO, USA) were additionally used in the fluorescence measurements. The concentration of the NaCl solution was 10 mM. The AO solution was prepared at a concentration of 1 mM by dissolving the appropriate quantity in ethanol.

### 3.2. Spectroscopic Methods

#### 3.2.1. UV-Vis Spectroscopy

The use of ultraviolet spectroscopy (UV) enabled the characterization of the interaction between the phthalimide derivatives and ctDNA. The Jasco V750 spectrophotometer (Jasco, Tokyo, Japan) recorded the spectra with a wavelength range of 200–350 nm with a 0.2 unit increment and the speed scan was 400 nm/min. Each sample was placed in a quartz cuvette with an optical path length of 10 mm, and an automatic baseline correction was applied.

The series of UV spectra was recorded at varying molar ratios of the ingredients. It was allowed to determine the apparent association constants K by the standard procedure of Benesi–Hildebrand [16]. The data were fitting to the linear function according to Equation (1):

(1) $$\frac{1}{A_{obs}-A_{0}}=\frac{1}{A_{c}-A_{0}}+\frac{1}{K\left(A_{c}-A_{0}\right)c_{a}}$$

where *A_obs_*—the compound solution absorbance with different ctDNA content; *A*_0_—the compound solution absorbance; *A_c_*—the alkaloid/ctDNA complex absorbance; and *c_a_*—the concentration of ctDNA. The correlation between the thermodynamic parameters and the apparent constant describes the van’t Hoff relation. The change in standard free Gibbs energy (ΔG) was calculated according to the following Equation (2) [37]:

(2) ∆G=−RTlnK

where *R*—the gas constant 8.314 J·mol^−1^·K^−1^; *T*—the temperature 298 K; and K—the apparent association constant.

The final volume of the samples for the UV absorption spectroscopy measurements was 3 mL, obtained by mixing 25 μL of ctDNA, multiple of 0.25 molar equivalents of the compound in every step, and topped up with PBS.

#### 3.2.2. Circular Dichroism Spectroscopy

The circular dichroism spectra were obtained using a Jasco J-1500 magnetic circular dichroism spectrometer (JASCO International CO., Tokyo, Japan). The measurements were conducted at room temperature with a 10 mm path length in a quartz cuvette. The following measurement parameters were employed: The measurement range was 230–320 nm with a resolution of 0.1 nm and a scan speed of 100 nm/min. Each spectrum was obtained by averaging 20 accumulations, resulting in a spectrum representative of the entire measurement series. A solution of ctDNA was prepared by dissolving an appropriate quantity of the stock solution in phosphate buffer (pH 7.4), resulting in a concentration of 100 μM. Subsequently, the requisite quantities of the analyzed compound were introduced to the aforementioned sample, thus yielding 0.5 and 1 molar ratios of the phthalimide derivative to the macromolecule.

#### 3.2.3. Fluorescence Spectroscopy

A Cary Eclipse 500 spectrophotometer (Agilent, Santa Clara, CA, USA) was used to measure the fluorescence spectra.

This method employs the use of a marker that has been previously demonstrated to interact with ctDNA in a well-defined manner, namely acridine orange. It binds to the macromolecule by intercalation. To the sample containing ctDNA at a concentration of 50 μM and an equimolar amount of acridine orange, subsequent portions of the phthalimide derivative were added and the spectrum was recorded at the 471 excitation line, in the range of 510–650 nm. The measurements were performed for the following molar ratios of ctDNA to phthalimide analog: 1:0, 1:0.5, 1:1, 1:1.5, and 1:2. Based on the obtained spectra, the percentage of AO replacement by the tested compound was calculated according to Equation (3):

(3) $$\%Re=\frac{F_{0}-F}{F_{0}}\cdot 100\%$$

where F_0_ is the fluorescence intensity of the AO/ctDNA complex, and F is the fluorescence intensity upon adding the studied compound.

Fluorescence spectroscopy was also used to study the electrostatic interactions in the analyzed systems **A**-, **B**-, and **C**/ctDNA. The NaCl solution was added in volumes from 0 to 240 μL to the solution containing equimolar amounts of ctDNA and a phthalimide derivative at concentrations of 50 μM. The samples were excited at wavelengths of 237 nm, 232 nm, and 243 nm for compounds **A**, **B**, and **C**, respectively [3]. The spectra were recorded in the range of 250–450 nm.

### 3.3. Molecular Modeling

The docking studies were performed using AutoDock Tools 1.5.6 [38] and the AutoDockVina 1.1.2 script [39]. The 3D structures of the compounds were optimized using the Gaussian 2016 C.01 software package [40] with the B3LYP/6-31+G (d.p) basic set. The B-DNA-1vzk [30] structures were used. For the ligands, rotatable bonds were assigned, non-polar hydrogens were merged, and partial charges were added. For the crystal structure of DNA, co-crystallized molecules and water were removed. Kollman partial charges and non-polar hydrogens were added. The docking protocol was first validated by self-docking the co-crystallized ligand.

The MD simulation was carried out with the Gromacs 2021.2 software [41] using an Amber 19SB force field [42,43]. The ligand topology was prepared using the CHARMM-GUI server [44,45]. The TP water model KCl (0.15 M) for neutralization was applied. The energy of the system was minimized and then the system was equilibrated for 125 ps. Next, the MD simulation was performed for 50 ns with a step of 2 fs, considering a constant pressure of 1 bar and a constant temperature of 303 K. The binding free energy ΔG_bind_ was calculated using the gmx_MMPBSA tool v.1.6.1 [46]. For the visualizations of the docking studies, Discovery Studio Visualizer v.24.1 was used. The RMSD plots were made using OriginPro 2024 v.10.1.

## 4. Conclusions

The present paper describes the interaction of four phthalimide derivatives, designated **A**, **B**, **C**, and **D**, with a ctDNA molecule. Both spectroscopic analytical methods and in silico methods were employed. The results demonstrated that the tested compounds bind to the macromolecule in the minor groove. The UV-Vis spectroscopy showed that the most stable complex was formed in the C/ctDNA system, with a molecule comprising three fluoride substituents. The apparent constant calculated for this system was equal to 1.28 × 10^4^ dm^3^·mol^−1^. For the remaining analyzed systems, these values were an order of magnitude lower. The standard Gibbs free energy for all the analyzed systems was negative and of the order of 10^4^. Furthermore, circular dichroism spectroscopy demonstrated that the molecules under investigation did not exert a notable influence on the spatial structure of the DNA. Electrostatic interactions were excluded using fluorescence spectroscopy, and in the studies with the acridine orange marker, the intercalation mode of binding of the tested compounds to the macromolecule was also excluded. The molecular modeling confirmed the mechanism of interaction suggested by the experimental results. It is noteworthy that phthalimide derivatives interact with deoxyribonucleic acid in a manner analogous to the pharmaceutical agents in this class, namely thalidomide, lenalidomide, and pomalidomide.

## Figures and Tables

**Table 1:** The linear fitting parameters of the Benesi–Hildebrand equation were determined by the absorbance changes.

Complex	Intercept	Slope	R^2^
**A**/ctDNA	0.3595	2×10^−4^	0.9802
**B**/ctDNA	0.2899	1×10^−4^	0.9941
**C**/ctDNA	0.8928	7×10^−5^	0.9899
**D**/ctDNA	0.3937	8×10^−5^	0.9987

**Table 2:** The binding parameters: the percentage of hyperchromicity, the apparent constant (K), and the standard Gibbs free energy (ΔG) for compound **A–D** interaction with ctDNA.

Complex	% Hyperchromicity	K [dm^3^·mol^−1^]	ΔG [J·mol^−1^]
**A**/ctDNA	58.75	1.80×10^3^	−1.86×10^4^
**B**/ctDNA	51.76	2.90×10^3^	−1.98×10^4^
**C**/ctDNA	76.40	1.28×10^4^	−2.34×10^4^
**D**/ctDNA	70.84	4.86×10^3^	−2.10×10^4^

**Table 3:** The position and the intensity change in the bands observed in the CD spectra with the increase in the concentration of the tested phthalic acid imide derivatives for the **A**-, **B**-, **C**-, and **D**/ctDNA systems.

Compound/ctDNA Molar Ratio	Δε_245nm_ [mdeg]	Δε_280nm_ [mdeg]
Compound **A**		
0	−8.08	7.91
0.5	−7.63	7.57
1	−7.23	7.29
Compound **B**		
0	−6.83	7.52
0.5	−6.50	7.12
1	−6.25	6.87
Compound **C**		
0	−8.13	7.43
0.5	−7.65	7.43
1	−7.15	7.09
Compound **D**		
0	−8.05	7.93
0.5	−7.65	7.52
1	−7.22	7.32

**Table 4:** The percentage of replacement for exchanging AO by **A**, **B**, **C**, and **D** phthalic acid imide derivatives in ctDNA complexes for a two-fold excess of the tested compounds.

Compound	% of Replacement
**A**	5.70%
**B**	11.82%
**C**	9.84%
**D**	11.33%

## Data Availability

Data are contained within the article.
